# Poor Law Topics

**Published:** 1906-01-13

**Authors:** 


### POOR LAW TOPICS.

### PAUPER PATIENTS IN ISOLATION HOSPITALS.

An important case dealing with the liability of a Union with regard to paupers treated in an isolation hospital outside its bounds has recently been decided by Mr. Justice Bray at the Manchester Assizes. Some children belonging to the Chorlton Union had been boarded out at the Bishop of Salford's schools in the urban district of Tottington, which is within the Bury and District Joint Hospital district. Scarlet fever broke out in the school, and some of the Chorlton children were attacked by it. The medical adviser of the school, Dr. Poole, who was also Medical Officer of Health for the district, recommended their being sent to the "Florence Nightingale" hospital at Bury, and, the Lady Superintendent of the school acquiescing, they were removed there immediately, and received without question. The Lady Superintendent communicated with the Clerk to the Chorlton Guardians, who merely acknowledged receipt of the letter, expressed regret at the outbreak, and said that in the meantime no more children should be sent from the workhouse to the school. When the illness was past the hospital authorities came upon the Chorlton Board for payment of the cost of the treatment given to the children chargeable to the Union. This demand the Guardians refused to pay, on the ground that it was not by their orders that the children were admitted to the hospital. The question then was whether the Lady Superintendent of the school had the authority of the Guardians to pledge their credit for the expenses of treatment and maintenance of the children while they were in the hospital. It was argued that as the Union authorities knew that the Superintendent had sent the children to the hospital, and had not protested against her doing so, she had pledged their credit for the inevitable expense. But Mr. Justice Bray held that as there had been no expressed promise to pay, and as the letter from the Clerk of the Guardians contained no mention of the fact—though duly communicated—that the children had been taken to the fever hospital, there was no implied promise that the expenses would be met by the Guardians. Moreover, the Clerk to the Hospital Board wrote to the Clerk of the Guardians : "It is proposed to charge your Board for the maintenance of the children," and Mr. Justice Bray held that the words "it is proposed to charge" showed that there was no agreement as to payment. In consideration of these facts, he held that the Guardians were not liable for the cost incurred by the authorities of the hospital in respect of the Chorlton children. The legal accuracy of the judgment is undeniable, but one cannot but hold that it is rather an unfortunate one. For it may result in the refusal of the authorities of isolation hospitals to receive pauper patients belonging to a Union outside the district for which they are intended to cater, and it is easy to conjecture how much suffering such a refusal may cause. Had the children not been received into the "Florence Nightingale" hospital, they would have had to be removed to some institution within their Union, 15 miles away, and the results of such a journey to patients suffering from scarlet fever might have been serious. Under the circumstances the Guardians ought to have considered the advantage to the children of being received into a convenient hospital, and have paid the costs thereby incurred. As, however, the law declares that they are free from liability, it would seem necessary for the Local Government Board to make an authoritative pronouncement on the duties and liabilities of both parties in such a juncture.

